# Condylar Asymmetry in Children with Unilateral Posterior Crossbite Malocclusion: A Comparative Cross-Sectional Study

**DOI:** 10.3390/children9111772

**Published:** 2022-11-18

**Authors:** Alessandro Tortarolo, Rossana Rotolo, Ludovica Nucci, Michele Tepedino, Vito Crincoli, Maria Grazia Piancino

**Affiliations:** 1 Department of Surgical Sciences, Dental School, University of Turin, Via Nizza 230, 10126 Turin, Italy; 2 Multidisciplinary Department of Medical-Surgical and Dental Specialties, School of Medicine and Surgery, University of Campania Luigi Vanvitelli, Via Luigi De Crecchio 6, 80138 Naples, Italy; 3 Department of Biotechnological and Applied Sciences, University of L’Aquila, Piazzale Salvatore Tommasi 1, Blocco 11, 67010 L’Aquila, Italy; 4 Department of Basic Medical Sciences, Neuroscience and Sense Organs, University of Bari, Policlinico, Piazza G. Cesare 11, 70121 Bari, Italy

**Keywords:** unilateral posterior crossbite, malocclusion, facial asymmetry, radiography, panoramic, dentition, mixed

## Abstract

Unilateral posterior crossbite (UXB) is a common, severely asymmetric malocclusion, characterized by maxillary hypoplasia and masticatory dysfunction. The aim of this research is to evaluate the asymmetry of mandibular condyles and rami in children with UXB. This comparative cross-sectional study included 33 children with UXB (girls = 15, boys = 18; mean age ± SD = 8.0 ± 1.3 years.months]) and 33 age- and gender-matched controls (girls = 15, boys = 18; mean age ± SD = 8.4 ± 1.3 years.months]). Pre-treatment OPGs were analyzed by comparing the height of condyles and rami between the sides using the method by Habets et al. (1988); the result was considered significant if the degree of asymmetry was >6%. Children with UXB showed a significantly increased asymmetry of mandibular condyles (mean ± SD = 10.7% ± 9, *p* < 0.001), but not of rami (mean ± SD = 1.9% ± 2.3), compared to controls. The rami did not show significant asymmetry in either group. The presence of an increased condylar asymmetry index in a developing patient with unilateral posterior crossbite is a sign of altered skeletal growth and should be considered in the diagnostic process and treatment plan.

## 1. Introduction

Unilateral posterior crossbite is a severe asymmetric malocclusion characterized by an inverse occlusal relationship between one or more posterior teeth on one side of the dental arches so that one or more maxillary buccal cusps are positioned in the central fossae of the mandibular teeth [1]. Many different etiological factors may be associated with unilateral posterior crossbite, including functional alterations (e.g., oral breathing, atypical swallowing, and oral habits such as thumb sucking), congenital disease (cleft lip and palate), systemic conditions (dystrophies, metabolic disorders, early childhood infections, and trauma), and local conditions (dental agenesia, early tooth extraction) [2]. However, the most important factors associated with the presence of unilateral posterior crossbite are functional and skeletal asymmetry and maxillary hypoplasia, the latter of which is often accompanied by concomitant mandibular hyperplasia [3,4,5]. 

Posterior crossbite is a common malocclusion, ranging from 4% to 23% of the orthodontic population, with more than half of posterior crossbites being unilateral [5,6,7]. In a recent meta-analysis, its worldwide prevalence in the mixed dentition was estimated to be 8% [8]. Although it may be present already in the deciduous dentition, before the age of 6, it is more common after the eruption of the first permanent teeth. In general, it is not self-correcting and requires early orthodontic treatment [5]. If left untreated, it may be associated with irreversible dentoalveolar and skeletal asymmetric alterations [4,9,10,11,12]. 

Even though this malocclusion is diagnosed based on dental malposition, it may more accurately be described as a neuromuscular syndrome [2]. Recent research has established that unilateral posterior crossbite is significantly associated with severe alterations in masticatory function (i.e., a higher proportion of reverse chewing patterns on the side of the malocclusion), especially when the patient is chewing a hard bolus [2]. Reverse mastication is accompanied by altered muscle coordination between the sides; in particular, the masseter muscle on the side of the malocclusion is significantly hypoactive, whereas the contralateral muscle shows compensatory hyperactivity [2]. Considering the young age of these patients, the functional imbalance between the sides has a significant, deleterious influence on the development of the central motor control of masticatory function [13]. These functional alterations are the basis of the asymmetric development of craniofacial structures, particularly the mandibular condyles. These structures are characterized by an adaptive type of growth, as they are capable of morphological changes in response to functional loads throughout the course of life [14,15]. Posterior crossbite patients have been reported to be at increased risk of developing temporomandibular joint disorders later in life [5,16]. Furthermore, masticatory alterations have been shown to be associated with neurological effects in laboratory animals, including reduced neurogenesis in the hippocampus and reduced memory and spatial orientation [17,18], and recently published epidemiological studies have shown that reduced mastication is associated with a higher prevalence of cognitive impairment in the elderly [19,20]. Therefore, the treatment of unilateral posterior crossbite should be aimed at correcting the dental malocclusion and masticatory dysfunction at the same time, thus re-establishing the functional balance between the sides as early as possible [2].

Orthopantomographic images (OPGs) are routinely acquired during pediatric dentistry and orthodontic evaluation because they provide crucial information on the patient’s dentition. Since Habets et al. [21] introduced a method to assess the symmetry of mandibular condyles and rami on OPG images, a few studies have applied it to unilateral posterior crossbite patients in the mixed dentition [22,23,24]. In the literature, there is a degree of agreement on the fact that unilateral posterior crossbite patients show increased condylar asymmetry between the sides [25,26]. However, one study did not include a control group [22] and one study did not find any significant difference between unilateral posterior crossbite and control patients [24]. Another study found a significant difference in condylar asymmetry between unilateral posterior crossbite and control patients, but presented high levels of condylar asymmetry in control subjects as well [23]. In a previously published paper, we described a highly increased level of condylar asymmetry between the sides in juvenile idiopathic arthritis patients, whereas control subjects showed remarkable symmetry [27]. Given the high prevalence of unilateral posterior crossbite and its functional implications, there is a need to research this issue further. 

The aim of this comparative cross-sectional study is to compare a group of unilateral posterior crossbite patients in the mixed dentition with a group of age- and gender-matched control subjects, following the method proposed by Habets et al. The null hypothesis (H0) for this study is that there is no significant difference in mandibular condyle and ramus symmetry between the groups.

## 2. Materials and Methods

### 2.1. Study Design

This comparative cross-sectional study included patients consecutively selected among those referred to the Department of Orthodontics, Dental School, University of Turin, between 2021 and 2022. Before entering the study, informed consent was obtained from all parents or legal guardians. The study was conducted in accord with the Declaration of Helsinki and with the approval of the Institutional Review Board of the University Hospital Company (Turin, Italy), protocol code 0069193.

Inclusion criteria were: (1) mixed dentition, (2) unilateral posterior crossbite. Exclusion criteria were: (1) previous orthodontic treatment, (2) presence of hereditary or congenital disease, (3) evidence of temporomandibular disorders (diagnosed following the DC/TMD criteria [28]), and (4) unavailability of pre-treatment orthopantomographic documentation. Patients with mild malocclusion and a strictly symmetrical molar class relationship were selected as a control group. 

Pre-treatment OPGs were checked for imaging quality, presence of mandibular condyles in the field of view, and absence of radiographical artifacts projecting over the structures of interest. OPGs that showed excessive rotation of the patient’s head, as evidenced by a discrepancy between the size of the most distal lower molar crowns’ horizontal dimension, or other defects were excluded. 

After initial case selection, unilateral posterior crossbite patients were individually matched by gender and age (with a tolerance of ±6 months) with control patients to ensure comparability of the study groups.

We initially included 84 patients; after age- and gender-matching, we excluded 18 patients and included 66 age- and gender-matched patients in the analysis, which is 12 more than required by power analysis: 33 patients with unilateral posterior crossbite (F = 15, M = 18; age = 8.0 (1.3) [years.months]) and 33 control patients (F = 15, M = 18; age = 8.4 (1.3) [years.months]). Table 1 illustrates the characteristics of the included patients. 

### 2.2. Measurements on OPGs

**Figure 1:**
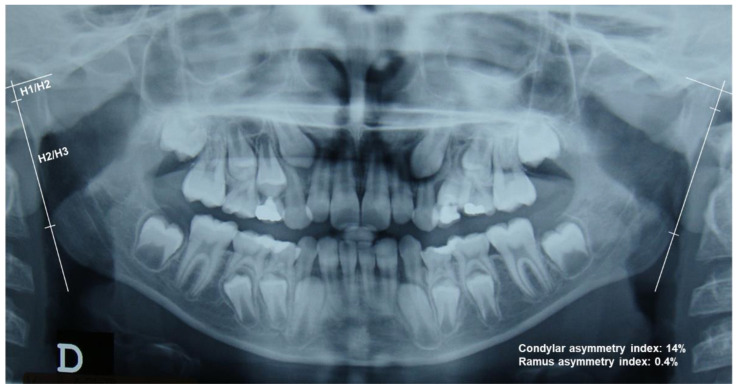
OPG of a mixed dentition patient with right unilateral posterior crossbite. The figure illustrates the use of the method by Habets et al., described in Materials and Methods. **H1/H2**: height of the condyle; **H2/H3**: height of the ramus. Note the condylar asymmetry between the sides. D refers to the right side.

The orthodontic diagnosis was confirmed clinically and on study casts for each included subject by two experienced orthodontists. As all patients in our department routinely undergo orthopantomographic evaluation before the beginning of orthodontic treatment, no additional radiological imaging procedures were necessary for this study, in accordance with the ALARA concept of radioprotection. For each patient included in this study, we collected the OPG from the first clinical case evaluation. The analysis of mandibular condyles and rami was performed following the method described by Habets et al. [21]. Briefly, as shown in Figure 1, the operator traced the outline of the mandible, including the condyles, on acetate paper by means of a pencil with a 0.5 mm tip. On each side of the outline of the mandible, a line tangent to the most lateral point of the ramus and to the most lateral point of the condyle (line V) was traced, followed by three perpendicular lines, conducted as follows: one tangent to the highest point of the condyle (line H1); one intersecting line V at the most lateral point of the condyle (line H2); and one intersecting line V at the most lateral point of the ramus (line H3). Next, the operator measured the distances H1/H2 and H2/H3 on each side with digital calipers. The operator that performed the tracings was blind, i.e., unaware of the patients’ allocation to the experimental groups. 

Therefore, the height of the OPG image of the condyle corresponded to the distance H1/H2 and the height of the OPG image of the ramus corresponded to the distance H2/H3. To assess the asymmetry between the right (R) and left (L) condyles and rami (asymmetry index), the formula │(R − L)/(R + L)│ × 100 was used. An asymmetry index ≤6% indicated that the left and right structures were to be considered symmetrical, whereas a result >6% was indicative of asymmetry. The 6% threshold accounts for slight rotations in patient positioning during the exposure of the OPG, resulting in asymmetrical enlargement of the structures of interest [21].

### 2.3. Statistical Analysis

Data are shown as mean (standard deviation). Normality of variable distributions were assessed with the Shapiro-Wilks test and the appropriate parametric or non-parametric test was chosen accordingly. All tests were two-tailed.

We performed a preliminary power analysis to estimate the minimum sample size necessary to detect a difference. Based on preliminary results and previously published reports, we assumed likely mean values of condylar asymmetry index to be 10% (10) for the posterior crossbite group and 4% (4) for the control group, and estimated a total sample size of 52 to detect a difference in condylar asymmetry index of 6%, with alpha = 0.05 and a power value of 0.8. 

To evaluate the error rate of the method, a subset of 15 OPGs was randomly selected and the tracings were repeated by the same author; the two sets of measurements were compared with a Wilcoxon signed-rank test, and the intraclass correlation coefficient (ICC) was assessed with a two-way mixed effects model. After age- and gender-matching, we compared mean age in the two groups with a Student’s t-test. The proportion of asymmetric individuals in the two groups was compared with a Fisher’s exact test. A between-group comparison of the asymmetry index was performed using a Mann-Whitney test and stratified by sex. The statistical significance level was set at 5%. The software STATA 17 (StataCorp, 4905 Lakeway Drive, College Station, TX 77845, USA) was used for all statistical analyses.

## 3. Results

There was no significant difference in age, body weight, height, or BMI between the groups. The evaluation of method error showed that there was no significant difference between the two sets of tracings; ICC calculations showed that the average consistency of agreement was 0.89 (CI: 0.67–0.96) for the right condyle, 0.92 (CI: 0.77–0.97) for the left condyle, 0.97 (CI: 0.90–0.99) for the right ramus and 0.97 (CI: 0.90–0.99) for the left ramus, indicating an excellent degree of reproducibility. 

**Figure 2:**
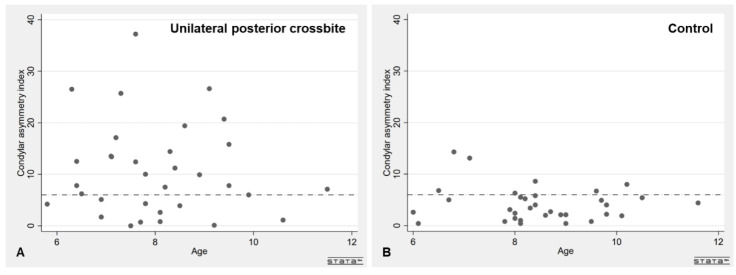
Scatter plots showing the patients’ age on the *X*-axis and the condylar asymmetry index on the *Y*-axis. The dot-and-dash line corresponds to a condylar asymmetry index of 6%, the threshold value for asymmetry between the sides. (**A**) unilateral posterior crossbite patients; (**B**) control patients.

**Figure 3:**
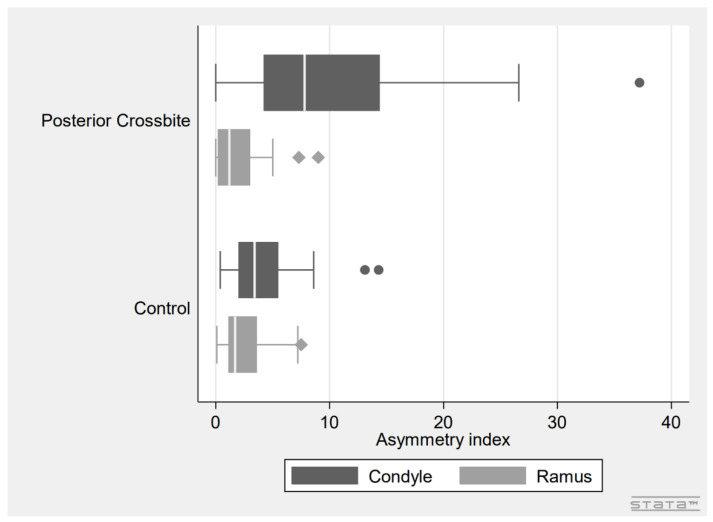
Box plot showing the asymmetry index of condyles and rami in unilateral posterior crossbite patients and controls. Dots and diamonds represent outliers (dots: condyle; diamonds: ramus). Note the significantly higher condylar asymmetry index in unilateral posterior crossbite patients.

The proportion of patients with asymmetry of the condyle was significantly higher in the unilateral posterior crossbite group compared to controls (*p* < 0.0001), whereas there was no significant difference in the proportion of patients with asymmetry of the ramus between the groups. In the unilateral posterior crossbite group, the mean asymmetry index was 10.7% (9.0) for the condyles and 1.9% (2.3) for the rami, whereas in the control group the mean asymmetry index was 4.2% (3.3) for the condyles and 2.5% (2.0) for the rami. The mean asymmetry index was significantly higher in unilateral posterior crossbite patients compared to controls (*p* < 0.001). The mean asymmetry index for the rami was below the 6% threshold in both groups. There was no difference in mean asymmetry index of the condyles or rami after the groups were stratified by sex. See Table 2, Figure 2 and Figure 3.

According to the results, the null hypothesis (H0) could be rejected for the mandibular condyle, but not for the ramus. 

## 4. Discussion

In this comparative cross-sectional study, we evaluated the asymmetry between mandibular condyles and rami in mixed dentition unilateral posterior crossbite patients and age- and gender-matched controls. The results showed a significantly increased asymmetry index for the condyles in unilateral posterior crossbite patients (*p* < 0.001), reflected in a significantly increased proportion of unilateral posterior crossbite patients with asymmetric condyles (*p* < 0.0001), compared to controls (Figure 3).

The mandibular outline in OPGs does not accurately reproduce the morphology of the condyles, and the method by Habets et al. [21] only provides an index of the difference in height between the condyles on the two sides. The vertical component of the structures that appear in OPGs is less subject to distortion in the posterior regions compared to the anterior ones, which makes this method sufficiently accurate. As OPGs are routinely acquired during pediatric dentistry and orthodontic evaluations, this method does not require any additional exposure to ionizing radiation or sophisticated equipment to be performed, characteristics that make it suitable for routine evaluation in the clinical setting. 

The temporomandibular joint is an important growth site during post-natal development. The mandibular condyle is responsive to mechanical stimuli and exhibits an adaptive (or compensatory, in pathological conditions) pattern of growth, [29]. At birth, the temporomandibular joint is completely immature; however, it undergoes important changes during growth, following the development of its function. At the breast-feeding stage, its flattened morphology reflects the sliding action required for efficient sucking. With the eruption of the deciduous dentition, it becomes steeper and the condyle relocates away from the occlusal plane [30,31]. Condylar adaptation or compensation may continue over the course of life, albeit at a slower pace, in response to ever-changing occlusal conditions [32].

**Figure 4:**
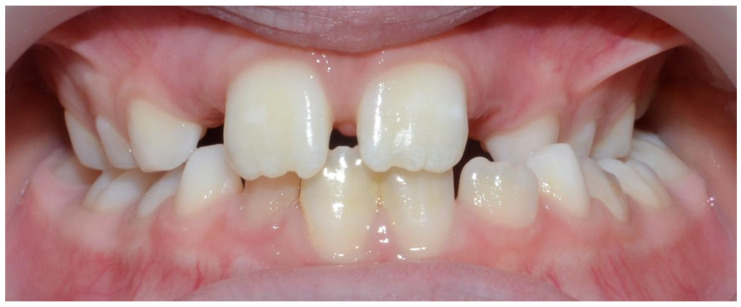
Unilateral posterior crossbite patient in the mixed dentition. Note the asymmetry of the upper and lower jaws.

The presence of unilateral posterior crossbite (Figure 4) entails an important masticatory dysfunction, manifesting as a significantly increased prevalence of reverse chewing patterns on the side of the malocclusion. This asymmetrical condition alters the balance of functional activity between the sides, with significant hypoactivity of masticatory muscles on the affected side and compensatory hyperactivity on the contralateral side. Functional activation is the main driver of the development of craniofacial structures [33,34]; this is especially true of the temporomandibular joint because of its capacity for adaptive growth. Clearly the presence of a severe asymmetrical malocclusion such as unilateral posterior crossbite has the potential to disrupt the fine balance of development between the sides, at the expense of the affected one.

In this study, we found a significant association between the presence of unilateral posterior crossbite and an increased condylar asymmetry index. This is in accordance with the few previous reports in the literature that indicate a condylar asymmetry index of approximately 10–12% in this malocclusion [22,23,24]. Likewise, we did not find any significant association between unilateral posterior crossbite and ramus asymmetry index, which was on average well below the 6% threshold. This is congruent with the different patterns of growth that characterize the condyle (adaptive growth) and the ramus (more genetically driven) [33]. Our study, on the contrary, found that the condylar asymmetry index in control patients (selected with a strictly symmetrical molar class) was on average very low, indicating the absence of relevant skeletal asymmetry between the sides. 

It is interesting to compare the average condylar asymmetry index of patients with juvenile idiopathic arthritis (JIA) and of patients with unilateral posterior crossbite [27]. In a previous study, we reported that the analysis of condylar asymmetry on OPGs in JIA patients showed on average a higher degree of asymmetry between the sides, in the range of 15%. This is consistent with the presence of condyle inflammation that leads to morphological rearrangements and reflects pathological alterations of the temporo-mandibular joint [27,35]. 

Furthermore, a few recent CBCT studies have shown three-dimensional asymmetrical alterations of the temporomandibular joints in unilateral posterior crossbite patients [10,36,37,38,39,40]. Even though these studies are very interesting, because they provide a more direct confirmation of the morphological alterations of mandibular condyles in unilateral posterior crossbite patients, the routine use of CBCT imaging on children should be viewed with caution.

The main limitation of this study is that it could not provide direct information about the morphological asymmetry of the mandibular condyles in unilateral posterior crossbite patients because condylar asymmetry was assessed using the orthopantomographic length of mandibular condyles instead of volumetric techniques. 

## 5. Conclusions

The presence of an increased condylar asymmetry index in a developing patient with unilateral posterior crossbite is a sign of altered skeletal growth and should be considered in the diagnostic process and treatment plan. The method used in this study takes advantage of routinely acquired OPGs and does not require additional exposure to ionizing radiation, which makes it very suitable for younger patients. Considering the high prevalence of condylar asymmetry in unilateral posterior crossbite, orthognathodontic correction of this malocclusion should be carried out early and should be aimed at restoring the skeletal and functional balance between the sides as well as treating the dental malposition.

## Figures and Tables

**Table 1:** Characteristics of included subjects.

	Unilateral Posterior Crossbite	Control
Age [years.months]	8.0 (1.3)	8.4 (1.3)
Molar class I	12	16
Molar class II	4	17
Molar class III	2	-
Molar class—asymmetrical	15	-
Unilateral posterior crossbite—Right	21	-
Unilateral posterior crossbite—Left	12	-
Body Weight [kg]	30.8 (8.8)	31.4 (8.1)
Height [m]	1.3 (0.08)	1.31 (0.1)
BMI	16.7 (2.9)	16.9 (3)
Total [number]	33	33

**Table 2:** Results.

	Unilateral Posterior Crossbite	Control	*p*-Value
^†^ Asymmetric condyles [proportion]	73%	21%	*p* < 0.0001
^†^ Asymmetric rami [proportion]	6%	9%	n.s.
^‡^ Mean asymmetry index—Condyle	10.7% (9.0)	4.2% (3.3)	*p* < 0.001
^‡^ Mean asymmetry index—Ramus	1.9% (2.3)	2.4% (2.0)	n.s.

^†^ Fisher’s exact test. ^‡^ Mann-Whitney test.

## Data Availability

The data presented in this study are available on request from the corresponding author. The data are not publicly available due to privacy concerns.
